# The Dynamics of HCF-1 Modulation of Herpes Simplex Virus Chromatin during Initiation of Infection

**DOI:** 10.3390/v5051272

**Published:** 2013-05-22

**Authors:** Jodi L. Vogel, Thomas M. Kristie

**Affiliations:** National Institute of Allergy and Infectious Diseases, National Institutes of Health, Bld. 33, 33 North Drive, Bethesda, MD 20892, USA; E-Mail: jvogel@niaid.nih.gov

**Keywords:** herpes simplex virus, chromatin, HCF-1, Setd1A, latency, histone demethylase

## Abstract

Successful infection of herpes simplex virus is dependent upon chromatin modulation by the cellular coactivator host cell factor-1 (HCF-1). This review focuses on the multiple chromatin modulation components associated with HCF-1 and the chromatin-related dynamics mediated by this coactivator that lead to the initiation of herpes simplex virus (HSV) immediate early gene expression.

## Abbreviations

HCF-1: host cell factor-1
FHL2: four and a half limb domain protein-2
GABP: GA-binding protein
Oct-1: octamer binding protein-1
VZV: varicella zoster virus
ASF1: anti-silencing function protein 1
PCAF: histone acetyltransferase KAT2B
SWI/SNF: switch/sucrose non-fermentable
ISWI: Imitation SWI
TBP: tata-binding protein
Med: mediator
MLL: histone-lysine N-methyltransferase MLL1
LSD1: lysine-specific demethylase 1
JMJD2: Jumonji domain-containing protein 2
PHF8: PHD finger protein 8
CHD8: chromodomain helicase DNA binding protein 8
NURF: nucleosome remodeling factor
THAP: thanatos-associated domain-containing apoptosis-associated protein
PGC: peroxisome proliferator-activated receptor gamma coactivator
PRC: peroxisome proliferator-activated receptor gamma coactivator PGC-1-related coactivator

## 1. Introduction

Infection of a cell with herpes simplex virus (HSV) results in a complex and dynamic interplay between host and pathogen on numerous levels. The progression of successful infection leading to the production of progeny virus is dependent upon both circumventing host suppression mechanisms and utilization of host machinery to express viral immediate early (IE) genes and thus establish the initiation of infection. 

This review focuses on the viral and cellular factors that govern the expression of IE genes and specifically, the more recently recognized impact of chromatin and chromatin modulation machinery in determining initial events leading to IE gene expression.

## 2. Viral and Cellular Factors that Govern IE Gene Expression

**Figure 1:**
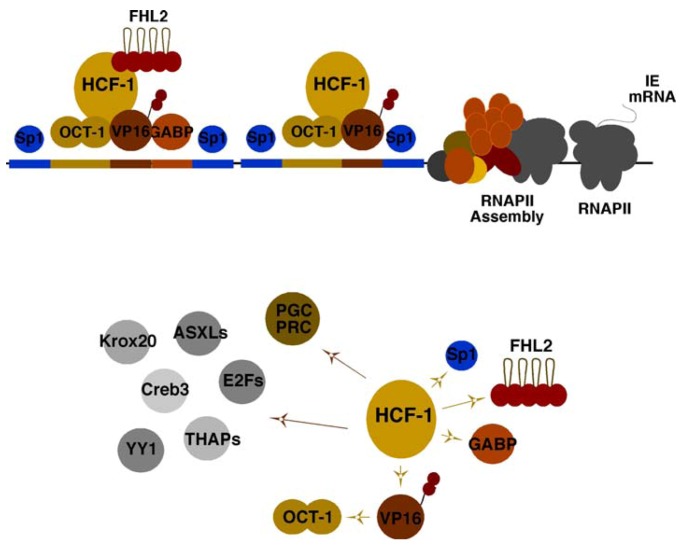
Elements and factors mediating herpes simplex virus (HSV) immediate early (IE) gene expression. (Top Panel) Viral IE promoter domains contain multiple reiterations of an enhancer core element that assembles the core complex (Oct-1, VP16, HCF-1), as well as sites for factors such as GABP and Sp1. FHL2 functions as a coactivator with HCF-1 for stimulation of IE gene transcription. (Bottom Panel) The essential coactivator HCF-1 interacts directly with transcription factors and coactivators that mediate IE gene expression (yellow arrows). A selection of other cellular factors that have been demonstrated to interact and require HCF-1 are illustrated (brown arrows).

The historical perspective of HSV viral IE gene expression is defined by the identification of IE regulatory elements and a complex set of transcription factors that cooperatively promote the expression of these genes (Figure 1, Top). One of the most critical elements is a reiterated sequence that functions as a classical enhancer module. This enhancer core element (ATGCTAATGARAT) is recognized by the cooperative assembly of the cellular Oct-1 POU domain factor (ATGCTAAT) and the viral encoded IE gene activator VP16 (α-TIF; GARAT) [1,2]. However, stable assembly of the enhancer core complex requires a third component, the cellular HCF-1 protein, which is now functionally recognized as a critical cellular coactivator/corepressor [3,4,5,6].

In addition to the enhancer core complex, the regulatory domains of each IE gene contains binding sites for the ETS protein GABP and Kruppel/Sp1 factors that synergistically amplify viral IE gene expression [7,8]. However, while expression of viral IE genes is enhanced by the VP16 enhancer complex, it is not entirely dependent upon this factor or upon the formation of the enhancer core complex. GABP and the coactivator FHL2 are examples of factors that have the potential to (i) stimulate IE reporter gene expression in the absence of VP16 and are (ii) regulated by multiple signaling pathways [9,10]. Thus, it is likely that many, as yet unidentified, activators and repressors regulate viral IE genes in distinct cell types and states.

## 3. The Essential HCF-1 Coactivator

While initially identified as a cellular component required for the stable assembly of the viral enhancer core complex and a determinant of VP16-mediated IE gene activation, the coactivator HCF-1 is an essential factor for IE gene expression. Depletion of HCF-1 results in abrogation of IE gene expression in contrast to the impacts of depleting other factors such as the enhancer core protein Oct-1 [11,12]. One rationale for this critical dependence is the striking direct interactions of HCF-1 with each of the identified primary transcription factors/coactivators (VP16, Sp1, GABP, FHL2) [5,9,10,13] and the requirement of this coactivator for mediating the activation potential of these components (Figure 1, Bottom). 

In addition to those that have been demonstrated to regulate viral IE gene expression, the critical nature of HCF-1 in the regulation of cellular programs has been demonstrated by its interaction and regulation of multiple DNA binding factors and coactivators [14,15,16,17,18,19] including: (i) cell cycle progression via modulation of the activities of the E2F family [20,21,22]; (ii) embryonic stem cell pluripotency and metabolism via E2F and THAP proteins [23,24,25]; (iii) metabolism and nuclear respiratory control via GABP and the coactivators PGC and PRC [26,27]. 

Importantly, the dependence of IE gene expression on HCF-1 suggested that the protein must mediate critical rate-limiting stage(s) in transcription.

## 4. HCF-1 and Multiple Chromatin Modulation Complexes

Studies that led to the elucidation of the role(s) of HCF-1 in governing chromatin modulation of alpha-herpesvirus (HSV and VZV) IE genes came from two intersecting directions; (i) the demonstration of the impacts of host-cell assembled chromatin on infecting viral genomes and (ii) the association of HCF-1 with multiple chromatin modulation components.

### 4.1. Chromatin Modulation of HSV Gene Expression

The first important clue to the regulation of HSV by chromatin was the observation that a virus encoding a mutant VP16 that lacked the protein’s activation domain exhibited accumulation of nucleosomes on the viral IE gene promoters [28]. This was consistent with a plethora of studies in general transcription biology using the VP16 activation domain that linked the activator to recruitment of basal transcription factors, mediator components (TBP, TFIIB, Med25), and chromatin modulation complexes (SAGA, SWI/SNF) [1,28,29,30,31,32,33,34]. However, the impact of VP16 on viral chromatin went largely ignored, due to the lack of substantial evidence that the infecting viral genome was subject to significant chromatin assembly and regulation during the lytic replication cycle.

Subsequently, however, it was clearly demonstrated that the viral genome was rapidly assembled into nucleosomal arrays mimicking host cell chromatin [35,36,37] (see Schang *et al.*, this issue). In addition, (i) histone chaperones were required for efficient HSV IE gene expression (HIRA) [38] and subsequent DNA replication (ASF1b) [39], and (ii) nucleosomes bearing marks characteristically associated with active chromatin were detected at viral gene promoters at appropriate times post infection [40,41,42]. With respect to chromatin remodeling complexes, while the viral activator VP16 can recruit the BAF (SWI/SNF) complex remodelers BRG/BRM, these proteins had no apparent impact on viral IE gene expression [43]. Rather, the ISWI component SNF2H was recruited to viral IE promoters and was important for IE gene expression [44]. 

Studies to identify acetyltransferases that might regulate viral chromatin concluded that p300, CBP, PCAF and GCN5 did not appear to play a significant role [45]. However, the circadian acetyltransferase CLOCK was shown to localize to sites associated with the infecting virus, was stabilized by infection, and was required for efficient viral gene expression [46,47] (see Roizman *et al.*, this issue). These data suggested that CLOCK might be important for acetylation of nucleosomes associated with the viral genome, although direct histone acetylation remains to be demonstrated. 

In addition to the investigation of host cell acetyltransferases that may regulate viral gene expression, the viral IE protein ICP0 has been implicated in promoting histone acetylation and preventing deacetylation, leading to a decreased level of stable nucleosomes on the viral genome [40,48,49,50]. Thus, while the acetyltransferase complexes that impact viral chromatin remain undefined to date, it is clear that both viral and cellular factors will contribute to the regulation of acetylation levels.

Most importantly, the combined studies clearly pointed to the requirement for various histone/ chromatin modulation machinery for efficient viral IE gene expression and progression of infection. With respect to the characteristics of the chromatin associated with the viral genome at the onset of infection, several important studies demonstrated that marks characteristic of repressive chromatin (*i.e*., histone H3K9-methylation) were prevalent, likely as a result of cellular responses to the invading pathogen [51,52,53]. As described below, this represents a critical dynamic interplay between host and pathogen, leading to either (i) suppression or (ii) circumvention of repression and progression of infection. 

### 4.2. HCF-1 Couples Removal of Repressive Histone H3K9-Methylation with Deposition of Activating Histone H3K4-Methylation

Concomitant with the developing hypothesis that the infecting HSV genome was subject to the regulatory impacts of assembled chromatin, the critical IE transcriptional coactivator HCF-1 was identified as a component of the Setd1A and MLL histone H3K4 methyltransferase complexes [54,55,56,57,58]. As H3K4-methylation is the canonical activating mark recognized by complexes that promote transcription, these studies were the first indication that the role(s) of HCF-1 in mediating gene expression, and presumably IE gene expression, were via chromatin modification/modulation. 

Subsequently, two studies indicated that the HCF-1-associated methyltransferases were important for regulation of viral gene expression. In these studies: (i) depletion of Setd1A resulted in impaired HSV gene expression [41]; (ii) Setd1A/MLL1 were both recruited to the related alpha-herpesvirus Varicella Zoster Virus (VZV) IE gene promoter in an HCF-1-dependent manner [52]; and (iii) the resulting H3K4-methylation was correlated with viral activator-mediated IE induction [41,52]. Interestingly, as noted above, in very early stages of infection or in HCF-1 depleted cells, nucleosomes bearing the dominant repressive H3K9-methylation mark were readily detected on the viral genome and specifically on the promoter domains of the viral IE genes [51,53]. This led to the hypothesis that initial infection triggered cell-mediated deposition of repressive chromatin on the viral genome that required removal by specific H3K9 demethylases in order to promote viral gene expression.

**Figure 2:**
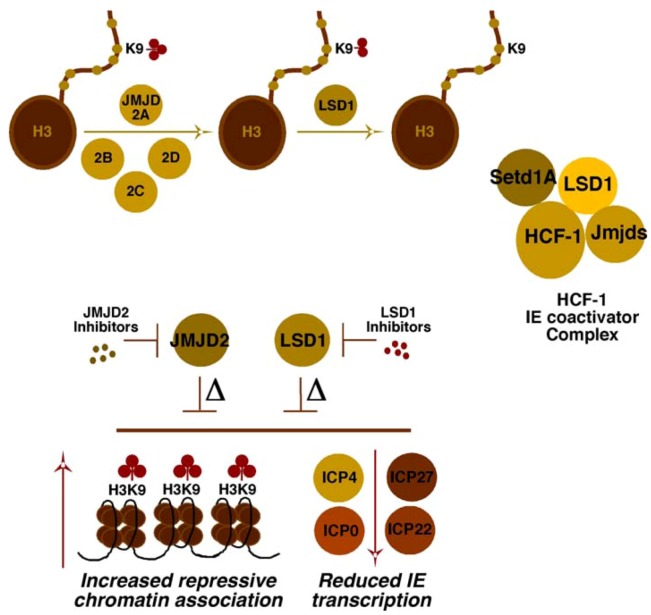
Cooperating activities of the histone demethylases, LSD1 and JMJD2(s), for derepression of HSV IE genes. The JMJD2 family of histone demethylases can remove histone H3K9-me3 but require the cooperating activity of a second demethylase (*i.e*., LSD1) to remove H3K9-me2/1. The HCF-1 coactivator complex identified contains both a histone H3K4-methyltransferase (Setd1A) and H3K9-demethylases (JMJD2/LSD1) to promote viral IE transcription. Depletion (∆) or inhibition of the catalytic activity of either LSD1 or the JMJD2 family results in reduced viral IE gene transcription and increased assembly of repressive chromatin on IE gene promoter domains.

Investigations into the components that might play roles in reversing repressive chromatin marks associated with the viral genome revealed two sets of interdependent H3K9 demethylases (Figure 2). Strikingly, both the family of JMJD2 proteins [59] and LSD1 [60] were shown to be important to limit the accumulation of repressive H3K9-methylation on the viral genome [51,61,62]. Either depletion of these proteins or inhibition of their catalytic activities with small molecule inhibitors blocked IE expression and resulted in enhanced histone and H3K9-methylation associated with the viral IE promoter domains. With respect to the role of HCF-1 in this conversion from repressive to activating methylation, an HCF-1 complex was identified that contained both the required demethylases (JMJD2/LSD1) and methyltransferase (Setd1A/MLL1) activities [51,62]. Thus, the recruitment of HCF-1 complex(es) by the enhancer core factors or via factors regulating IE genes suggested a combined potential to circumvent cell-mediated repression and promote IE gene transcription.

### 4.3. Multiple Chromatin Modulation Components/Complexes Associated with the HCF-1 Coactivator

**Figure 3:**
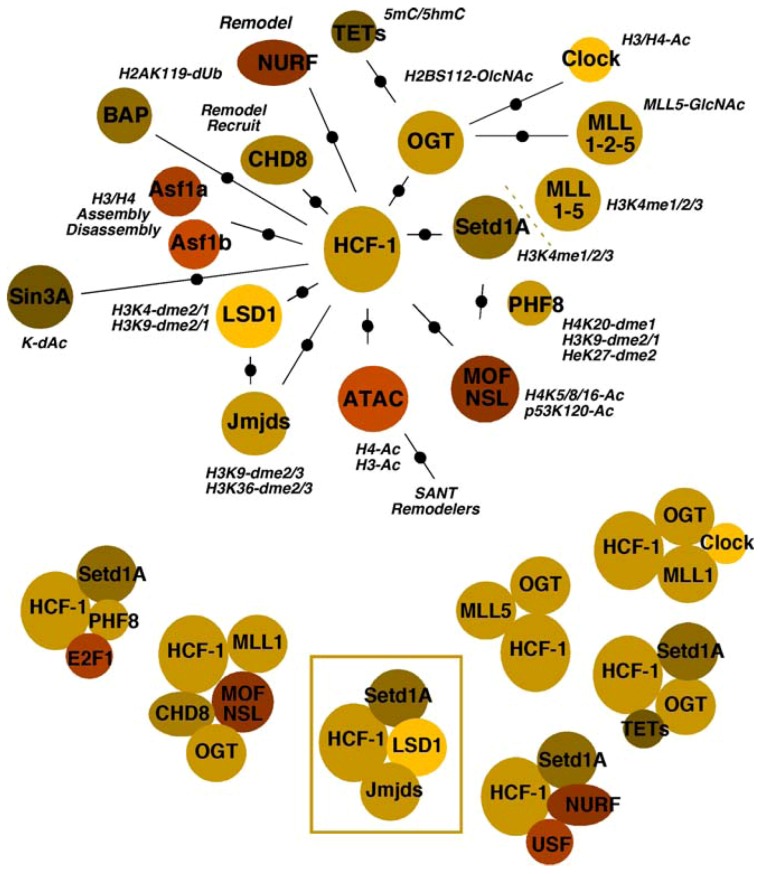
Multiple associations and complexes of the coactivator HCF-1 with chromatin modulation components. The multiple interactions or associations of chromatin modulation machinery are shown relative to HCF-1. Refer to the text for appropriate description and references. (Top Panel) The activities of components/complexes are noted (Ac, acetylation; GlcNAc, O-linked N-acetylglucosamine; dme, demethylation; dUB, deubiquination; dAc, deacetylation). (Bottom Panel) HCF-1 chromatin modulation complexes that have been clearly defined or identified in specific contexts are represented. The HCF-1 complex whose components are critical for viral IE gene expression is boxed.

In addition to the Setd1A/MLL1 H3K4 methyltransferases and the H3K9 demethylases LSD1/JMJD2(s), HCF-1 has been identified as a component of or has been associated with multiple chromatin modulation components. Whether these complexes/components are involved in the regulation of alpha-herpesviral chromatin has not yet been determined. It should be noted that the global state of HSV chromatin during various stages of infection is relatively uncharacterized and therefore, future studies may ultimately provide linkages between multiple HCF-1 complexes and modulation of HSV chromatin. Given the important nature of this coactivator for viral expression and the interplay of histone modification, recognition, and remodeling components that must occur during IE gene transcription, there is the potential that insights into control of viral chromatin may come from the studies of HCF-1 chromatin complexes. Therefore, relationships between HCF-1 and various chromatin components are shown in Figure 3 and detailed below.

#### 4.3.1. Histone Methyltransferases and Demethylases

In addition to the demonstrated roles of JMJD2s/LSD1 in removing repressive H3K9-methylation coupled with Setd1A/MLL1 in promoting H3K4-methylation of IE promoter-associated nucleosomes, HCF-1 is a component of complexes containing multiple members of the MLL family (MLL1, MLL2, MLL5) [55,56,58,63,64]. Several lines of evidence indicate that, while the bulk of cellular H3K4-methylation is due to Setd1A, the MLLs are responsible for the control of some developmental (MLL1/2), cell cycle (MLL1) and signal-induced transcription (MLL3/4). 

With respect to cell cycle, HCF-1 interacts with E2F1 in conjunction with either MLL1 or Setd1A and the demethylase PHF8, resulting in H4K20-demethylation, H3K4-methylation, and cell-cycle-dependent transcription (G1/S transition) [21,22]. 

#### 4.3.2. Acetyltransferases and Deacetylases

Three sets of acetyltransferases have been associated with HCF-1. The MOF/NSL complex cofractionates with the HCF-1/MLL1 complex and the chromatin remodeling factor chromodomain helicase DNA binding protein 8 (CHD8) [65,66,67,68]. This complex thus couples the HMT with acetyltransferase activity targeting histone H4K5-8-16, marks characteristic of euchromatin. In addition, the NSL complex also modifies the activity of other targets including p53. The second complex, ATAC, contains the histone acetyltransferases GCN5/ATAC2 and appears to target histone H3/H4 for acetylation [69,70,71,72,73]. Interestingly, this complex also contains a stress-activated kinase (TAK1/MAP3K7), suggesting that the complex activity may be subject to specific induced signaling. Finally, the third complex is of specific interest as it is composed of HCF-1, MLL1, and the H3/H4 acetyltransferase CLOCK [74,75], a protein implicated in the regulation of HSV gene expression [46].

In addition to multiple acetyltransferase complexes, HCF-1 is also a component of the repressive Sin3A complex containing the histone deacetylases HDAC1/2 [57]. This complex, targeted via interactions with THAP transcription factors, mediates repression of differentiation-linked gene expression in embryonic stem cells [23,76]. In a parallel, but contrasting manner, an HCF-1/Setd1A complex is also recruited by THAP factors and is involved in stimulation of genes, leading to the induction of cell cycle and increased cell mass [24], also contributing to the maintenance of embryonic stem (ES) cell pluripotency.

#### 4.3.3. Chaperones

HCF-1 directly interacts with the histone H3/H4 chaperones Asf1a and Asf1b [39]. Although both chaperones associate with HCF-1, the functional significance may be distinct. Asf1a is a constitutively expressed chaperone that is involved in non-replicative histone assembly/disassembly. In contrast, Asf1b levels increase during the S phase of the cell cycle and the protein appears to preferentially function during DNA replication [77,78]. 

For HSV, HCF-1/Asf1b complexes are linked to viral replication factors, providing a unique mechanism for nucleosome reorganization coupled to viral DNA synthesis [39]. In contrast, one function of Asf1a appears to be non-replicative assembly of chromatin on the viral genome that may play a role in initial repression of IE gene expression. However, the role(s) of Asf1a in the regulation of viral gene expression throughout the lytic replication cycle is less clear, as depletion of Asf1a increased expression of viral IE genes but reduced viral replication and progeny [79].

#### 4.3.4. Remodelers

Two HCF-1-associated remodeling activities are components of multifunctional complexes. As noted above, the HCF-1/MLL1/MOF-NSL methyltransferase/acetyltransferase complex also contains CHD8, which recognizes histone H3K4-me2/3 [66]. While CHD8 has been characterized as a transcriptional repressor via remodeling and recruitment of histone H1 [80], it also plays a critical activating role in stimulating U6 RNAPIII transcription [81]. 

Interestingly, CHD8 is also a component of a second HCF-1 associated remodeling complex, NURF, which is recruited by the upstream binding factor (USF) along with HCF-1/Setd1A as components of chromatin boundary elements that prevent the spread of heterochromatin [82,83]. In addition to CHD8, NURF contains the important remodeler SNF2L that is characteristically involved in remodeling to create nucleosome-free regions in promoter domains.

#### 4.3.5. OGT

O-linked N-acetylglucosamine transferase, the sole enzyme responsible for O-GlcNAc modification of proteins at S/T residues, is a component of multiple HCF-1 chromatin-related complexes. Its activity is required in the HCF-1/MLL5 complex where O-GlcNAc modification of MLL5 significantly enhances H3K4-methyltransferase activity [63]. Similarly, the protein modulates the stability of the histone acetyltransferase CLOCK [84]. 

More recently, OGT has been recognized as a contributor to the histone code by modification of histones including histone H2BS112, thus promoting H2BK120-monoubiquination and enhanced H3K4-methylation [85,86]. 

OGT, in complex with HCF-1/Setd1A, is also targeted to CpG islands by the recently identified ten-eleven translocation (TET) proteins [87,88,89,90]. Occupancy of these regions correlates with the lack of detectable cytosine modifications. Interestingly, as the TET proteins are involved in active DNA-demethylation (conversion of 5-methylcytosine to 5-hyroxymethylcytosine), these complexes may represent a coupling of both DNA and histone modifications that promote transcriptional activation.

#### 4.3.6. BAP1

BRCA1-associated protein-1 is a deubiquitinase (DUB) with multiple targets including histone H2AK119-Ub, a repressive mark linked with histone H3K27-methylation, DNA-methylation, and histone H1 association [91,92]. Interestingly, the role of BAP1 and H2AK119-Ub remains unclear and may be dependent upon the particular context. As a component of the polycomb repressive complex PR-DUB, BAP1 appears to be required to balance the levels of H2AK119/H2AK119-Ub for effective PRC-mediated repression [93,94]. In contrast, deubiquination of H2A is correlated with gene activation and increased histone H3K4-methylation [92].

The roles of this protein and its DUB activity in association with HCF-1 may be significantly more complex. In addition to modulating the repressive H2A-Ub, BAP1 has multiple protein substrates, including HCF-1, OGT, and perhaps, YY1 [95,96,97]. BAP DUB activity is required for stabilization of OGT and may affect the interactions or functions of HCF-1 in cell-cycle regulation, thus having pleiotropic impacts. 

#### 4.3.7. The Potential for Multiple Roles of HCF-1 Complexes in Viral IE Gene Expression

It is now clear that epigenetic regulation of HSV infection is an important contributor to the determination of the outcome of infection and represents a supra-regulatory overlay beyond the contributions of individual DNA binding transcription factors [98,99,100,101]. Much remains to be determined with respect to the components required for modulation of chromatin associated with the viral genome during each stage of primary/lytic infection. However, the clear role(s) of the coactivator HCF-1 in mediating chromatin modulation during viral infection suggest that additional complexes associated with this protein may also contribute. It is important to note that while HCF-1 has been ascribed or linked to many chromatin modulation factors/complexes, it is also unclear whether there are core units, such as HCF-1/Setd1A/OGT, that recruit/interact transiently and sequentially with multiple factors to enact viral gene expression. Additionally, it is unclear as to the mechanisms by which specific HCF-1 complexes may be selectively recruited. For HSV IE gene expression, the viral activator VP16 preferentially interacts with the HCF-1/Set1 complex, as opposed to complexes containing Sin3A. Whether other regulatory factors recruit distinct HCF-1 complexes that are required or contribute to viral IE gene expression remains to be determined.

## 5. The Potential of Epigenetic-Based Antivirals

The recognition of important elements of chromatin-mediated control of viral infection coupled with the rapidly advancing development of specific chromatin modulation machinery inhibitors [102,103,104,105,106,107] leads to new potential for novel antivirals [108]. As a striking example, monoamine oxidase inhibitors originally developed to target MAO-A and -B for the treatment of severe depression, also inhibit the histone demethylase, LSD1 [109,110,111]. As noted, LSD1 is critical to the initiation of alpha-herpesvirus IE gene expression and in cultured cells, MAOIs and other developed specific LSD1 inhibitors block infection by preventing reversal of the accumulated repressive chromatin on the genome. 

**Figure 4:**
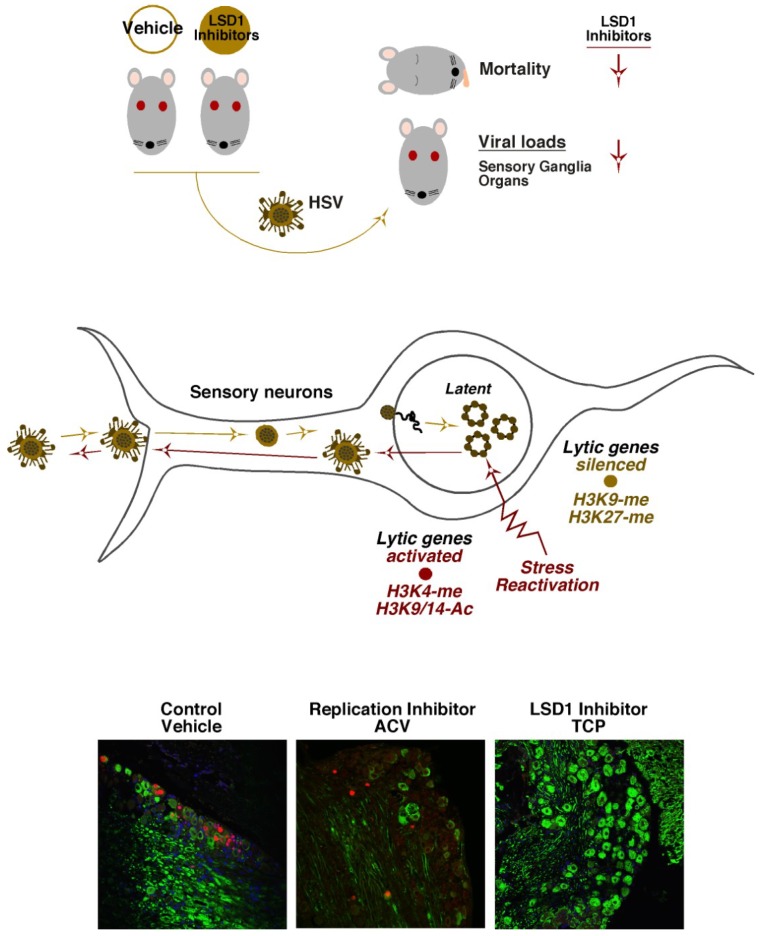
Inhibitors of the HCF-1 associated histone demethylases reduce primary infection and block viral reactivation from latency. (Top Panel) Mice treated with either Vehicle control or LSD1 inhibitors were infected with HSV. Mortality and viral loads were assessed at defined time periods post infection. LSD1 inhibitors reduce mortality and viral loads relative to control. (Middle Panel) HSV infection of sensory neurons results in the establishment of latency in which lytic genes are repressed. Stress-mediated reactivation of viral infection results in conversion of repressive chromatin marks to activating marks on viral lytic genes. (Bottom Panel) Latently infected trigeminal ganglia were explanted into culture to induce viral reactivation in the presence of control vehicle, ACV (acycloguanosine, DNA replication inhibitor), or the LSD1 inhibitor, TCP (tranylcypromine). Ganglia were sectioned and stained for neurofilament (green) and the viral lytic replication protein UL29/ICP8 (red) to mark neurons undergoing productive reactivation.

As a “proof of principle”, these compounds were tested for their ability to reduce an HSV primary infection in the mouse model system [51,61]. Treatment of mice prior to infection significantly reduced HSV mediated mortality and viral loads in sensory ganglia and other organs [61] (Figure 4, Top). While this represents an initial step in moving these compounds and related chromatin modulation inhibitors forward, it does indicate the potential for new inroads in antivirals that target the initiation stage of infection, prior to the expression of viral gene products. 

## 6. HCF-1 Chromatin Modulation Complexes and HSV Latency-Reactivation

HCF-1 chromatin modulation complexes play a dominant role in mediating lytic viral gene expression upon infection. Importantly, several lines of evidence also suggest that HCF-1 is an important component of the regulation that mediates the initiation of viral reactivation from latency.

HSV latency is established in neurons of sensory ganglia where the virus persists in a quiescent state in the absence of detectable viral IE gene expression [2] (Figure 4, Middle). Important and insightful studies have clearly correlated characteristic repressive marks (H3K9- and H3K27-methylation) associated with the viral lytic gene promoters during latency and activating marks (H3K4-methylation, H3-acetylation) with viral IE genes during reactivation [98,112,113,114,115,116,117,118,119,120] (see Bloom *et al.*, this issue). These studies were fundamental in establishing the potential control of latency-reactivation cycles by epigenetic mechanisms.

With respect to HCF-1, the protein is uniquely concentrated in the cytoplasm of sensory neurons but rapidly is transported to the nucleus of these cells in response to signals that promote viral reactivation from latency [121,122,123]. In addition, upon stimulation, HCF-1 can be rapidly detected occupying IE promoters of the viral genome [124]. These initial observations led to the hypothesis that HCF-1 is a component of the switch mechanism for HSV latency-reactivation cycles. Given the role(s) of HCF-1 in chromatin modulation during lytic infection, a model was proposed in which HCF-1 would be an integral part of the chromatin dynamics that must occur during the initiation of the reactivation process. The model was supported by studies in which inhibitors of either the HCF-1-associated demethylases, LSD1 or JMJD2(s), blocked the expression of viral IE genes and the production of viral progeny in induced latently infected sensory ganglia [51,61,62] (Figure 4, Bottom).

## 7. Concluding Remarks

Recently recognized, the role of chromatin modulation of alpha-herpesvirus infection represents an intricate and complex regulatory overlay. Intrinsically linked to viral-host interactions, chromatin presents a dynamic of repression/subversion of infection by the host cell. This is countered by mechanisms employed by the infecting virus to interface with the cellular chromatin machinery to promote viral gene expression and replication. A key player in this dynamic is the cellular chromatin regulator HCF-1 associated with multiple chromatin modulation components that the virus recruits at an early stage to promote lytic infection. Many stages of chromatin modulation and the enzymology that mediates it remain to be delineated for a true mechanistic view of this important aspect of viral infection. Understanding this dynamic and the key components could significantly increase the avenues for the development of novel antivirals with distinct advantages over present therapies.
